# Combined ^13^C-assisted metabolomics and metabolic flux analysis reveals the impacts of glutamate on the central metabolism of high β-galactosidase-producing *Pichia pastoris*

**DOI:** 10.1186/s40643-016-0124-6

**Published:** 2016-11-02

**Authors:** Ping Liu, Mingzhi Huang, Menglei Guo, Jiangchao Qian, Weilu Lin, Ju Chu, Yingping Zhuang, Siliang Zhang

**Affiliations:** State Key Laboratory of Bioreactor Engineering, East China University of Science and Technology, No.130, Meilong Road, Shanghai, 200237 China

**Keywords:** Glutamate, ^13^C metabolic flux analysis, Metabolomics, *P. pastoris*, Recombinant protein expression

## Abstract

**Background:**

*Pichia pastoris* is a popular recombinant protein expression system for its accessibility of efficient gene manipulation and high protein production. Sufficient supply of precursors, energy, and redox cofactors is crucial for high recombinant protein production. In our present work, we found that the addition of glutamate improved the recombinant β-galactosidase (β-gal) production by *P. pastoris* G1HL.

**Methods:**

To elucidate the impacts of glutamate on the central metabolism in detail, a combined ^13^C-assisted metabolomics and ^13^C metabolic flux analysis was conducted based on LC–MS/MS and GC–MS data.

**Results:**

The pool sizes of intracellular amino acids were obviously higher on glucose/glutamate (Glc/Glu). The fluxes in EMP entry reaction and in downstream TCA cycle were 50 and 67% higher on Glc/Glu than on Glc, respectively. While the fluxes in upstream TCA cycle kept almost unaltered, the fluxes in PPP oxidative branch decreased.

**Conclusion:**

The addition of glutamate leads to a remarkable change on the central metabolism of high β-galactosidase-producing *P. pastoris* G1HL. To meet the increased demands of redox cofactors and energy for higher β-galactosidase production on Glc/Glu, *P. pastoris* G1HL redistributes the fluxes in central metabolism through the inhibitions and/or activation of the enzymes in key nodes together with the energy and redox status.

**Electronic supplementary material:**

The online version of this article (doi:10.1186/s40643-016-0124-6) contains supplementary material, which is available to authorized users.

## Background

As a popular recombinant protein expression system, *P. pastoris* has been widely applied in pharmaceutical and chemical industries. This can be credited to its fine characteristics including the ability to perform post-translational modification (De Schutter et al. 2009; Hamilton et al. 2006), the accessibility for genetic manipulation (Cregg et al. 1993), the ability to obtain high cell density on simple and defined media (Cereghino et al. 2002), and the simple downstream harvesting process due to the capacity for heterologous secretion (Macauley-Patrick et al. 2005).

The high expression of recombinant proteins generates extra demands for precursors, energy, and redox cofactors, which are reflected in a reduced substrate uptake rate, lower specific growth (Cos et al. 2005), and decreased cell viability (Glick 1995). The phenomenon is known as metabolic burden (Glick 1995). To adapt to this metabolic burden, *P. pastoris* can readjust the central metabolism and restore its viability by triggering stress-response mechanisms (Heyland et al. 2011; Jordà et al. 2012).

Metabolic adaptations are related to changes in intracellular metabolite concentrations and metabolic fluxes. The high diversity of metabolites and complex structure of metabolic networks, however, hinder the precise quantification of metabolite concentrations and metabolic fluxes (Chen et al. 2011). ^13^C-assisted quantitative metabolomics and ^13^C metabolic flux analysis (^13^C-MFA) have become reliable methods for quantifying metabolite concentrations and metabolic fluxes, due to the improved precision from the use of ^13^C-labeled metabolites as internal standards in metabolomics analysis and from the use of ^13^C isotopic abundances as extra subjects when solving metabolic equations in metabolic flux analysis (Jordà et al. 2012; Niu et al. 2013; Wasylenko and Stephanopoulos 2015; Zamboni et al. 2009).

Some work has been done by combining these two methods to assess the effects of protein production on cell metabolism (Carnicer et al. 2012). Jordà and co-workers reported a higher trehalose pool size and trehalose cycle flux distribution which is related to the metabolic burden in the process of secreting protein *Rhizopus oryzae* lipase (Jordà et al. 2014).

In our previous study, a high β-galactosidase-producing strain *P. pastoris* G1HL and a low expression control strain *P. pastoris* GHL were engineered through controlling the initiation strength of the constitutive promoter *pGAP* (Qin et al. 2011). The addition of glutamate was found to be related to the improvement of β-galactosidase production (Nie et al. 2014). The increase of protein production through the addition of amino acids has been also observed in other studies (Görgens et al. 2005a, b; Heyland et al. 2011). However, the knowledge of the intracellular metabolism of *P. pastoris* after the addition of amino acids is limited. In the present work, in order to reveal the impacts of glutamate on the central metabolism of *P. pastoris* G1HL, a combined ^13^C-assisted quantitative metabolomics and ^13^C metabolic flux analysis was conducted. The pool sizes of intracellular metabolites and carbon fluxes in G1HL using glucose or glucose/glutamate as carbon sources were quantified. The energy and redox cofactor metabolisms were evaluated as well.

## Methods

### Strains

Two *P. pastoris* GS115 his^−^ (Cregg et al. 1993)-derived recombinant strains, the high β-galactosidase expression strain *P. pastoris* G1HL and the low expression control strain *P. pastoris* GHL, were constructed through changing the initiation strength of the constitutive promoter *pGAP* (Qin et al. 2011).

### Growth conditions in 5 L bioreactor

The batch cultivations were performed in a 5-L bioreactor (National Engineering Research Center for Biotechnology, Shanghai, China) with the working volume of 3 L. The media and growth conditions were the same as those described in our previous study (Nie et al. 2014). The fermentation medium contained 20 g/L glucose, 18.2 g/L K_2_SO_4_, 0.93 g/L CaSO_4_, 14.9 g/L MgSO_4_·7H_2_O, 4.13 g/L KOH, 26.8 mL/L H_3_PO_4_, 12 mL/L PTM1, and 1 mL/L antifoam. The PTM1 solution consists of 6.0 g/L CuSO_4_·5H_2_O, 0.08 g/L KI, 3.0 g/L MnSO_4_·H _2_O, 0.2 g/L Na_2_MoO_4_·2H_2_O, 0.02 g/L H_3_BO_3_, 0.5 g/L CoCl_2_, 20.0 g/L ZnCl_2_, 65.0 g/L FeSO_4_·7H_2_O, 0.2 g/L biotin, and 5.0 mL/L H_2_SO_4_. Specially, for the condition with glutamate, 5 g/L glutamate was added to medium initially. The temperature was controlled at 30 °C, and the initial aeration rate was controlled at 1 vvm. The dissolved oxygen (DO) was maintained above 30%, and the pH was controlled at 5.5 by adding 28% ammonium hydroxide (as nitrogen source). The pressure was maintained at 0.05 MPa using an automatic pressure control device (Burket, Germany). The off-gas was analyzed using a process mass spectrometer (MAX300-LG, Extrel, America). The parameters of aeration, agitation, dissolved oxygen, exhaust CO_2_, exhaust O_2_, OUR and CER, pH, pressure, temperature, and working volume of bioreactor were collected online by an in-house software BioStar. The cultivations were performed in duplicates.

### The ^13^C-labeling experiment

The preparation of ^13^C-labeling experiments was also the same as our previous study (Nie et al. 2014). A 250-mL bioreactor (National Engineering Research Center for Biotechnology, Shanghai, China) with the working volume of 150 mL was used. The initial glucose concentration was 20 g/L. 1.5 g labeled glucose was added into the reactor when the residual glucose concentration was about 10 g/L. The labeled glucose contained 80% [U-13C]-glucose (isotopic enrichment 99%, Cambridge Isotope Laboratories, Inc.) and 20% [1-13C]-glucose (isotopic enrichment 98–99%, Cambridge Isotope Laboratories, Inc.). The CO_2_ in the inlet air was eliminated by saturated NaOH solution. The labeling experiments were performed in two independent cultivation replicates.

### Quantitative analysis of biomass, glucose, glutamate, acetic acid, and β-galactosidase activity

The cell concentration was monitored by measuring the optical density at 600 nm. For dry cell weight (DCW) measurement, 5 mL broth was filtered using a predried and weighted micro-porous filter paper (Shanghai Diqing Filtration Technology CO., LTD Shanghai, China). After washing 3 times, the filter paper was dried at 95 °C for 2 h. The glucose kits (Shanghai Kexin Biotechnology Institute, China) were used for residual glucose concentration analysis.

Glutamate concentration was determined by a high-performance liquid chromatography (HPLC) system. 1 mL broth was centrifuged, and the supernatant was obtained. 20 μL supernatant was added to 100 μL boric acid buffer and then derivatized with 20 μL *O*-phthalaldehyde. Mobile phases (component A containing 40 mM NaH_2_PO_4_; component B containing methanol, acetonitrile, and ultrapure water mixed by 45:45:10) were applied at a flow rate of 2/mL/min using the Agilent Eclipe AAA 4.6 × 150 mm column. The absorbance detector was set at 338 nm. For the determination of acetic acid, an ion-exclusion column (Hi-Plex H, Agilent) was eluted at 50 °C with 10 mM H_2_SO_4_ at a flow rate of 0.5 mL/min and connected with an absorbance detector spectrophotometer at 210 nm.

The β-galactosidase assays were performed according to the protocol supplied in our previous study (Nie et al. 2014). One milliliter broth of yeast cells was disrupted, and β-galactosidase was detected by *Pichia pastoris* expression kit (Invitrogen). The broth was centrifuged (4 °C, 12,000 rpm, 10 min,) and cells were collected. 1 mL cell lysis buffer and 1 mL acid-washed glass beads (*D* = 0.5 mm) were added. The cells were broken by incubating in an ice bath for 30 s and then shaking for 30 s (repeated 8 times). The supernatant was collected after centrifugation.

For quantification of β-galactosidase, 50 μL cell extract was added into 1 mL preheated Z-buffer and the blank contained 50 μL cell lysis buffer. Then 0.2 mL 4 mg/mL ONPG was added to the samples. The reaction was conducted in water bath at 37 °C, and 0.5 mL 1 mol/L Na_2_CO_3_ was added to stop the reaction when the pale yellow appeared. Then, the optical density at 420 nm was measured using micro-plate reader (Thermo Scientific, America). The amount of enzyme that can hydrolyze 1 nmol ONPG to ONP per minute at 37 °C was defined as one unit of enzyme, i.e., 1 U. The mass of β-galactosidase was estimated upon the correlativity between the mass and activity of the pure β-galactosidase standards.

### Sampling and extraction of intracellular metabolites in ^13^C-labeling experiment

The labeled substrate was fed when the residual glucose concentration was about 10 g/L at the exponential growth phase. Once the labeled substrate was added, the samples were taken with a rapid-sampling setup (National Engineering Research Center for Biotechnology, Shanghai, China). Every time, about 1 mL broth was taken. During the first 5 min after labeling, ten samples were taken. Thereafter, several samples were taken until reaching the isotopically stationary state. The broth was directly injected into a tube with 15 mL pre-cooled quenching solution (60% methanol, −40 °C) (Carnicer et al. 2012) and then be filtered by a cellulose acetate fiber film (Millipore, USA).

The filter cakes were washed twice with deionized water. The intracellular metabolites were exacted with 30 mL 75% aqueous ethanol at 95 °C for 3 min (Canelas et al. 2009). The sample was centrifuged (12,000 rpm, 10 min), and the supernatant was collected. The supernatant was concentrated with a gas blowing concentrator.

### Labeling analysis of intracellular free amino acids by GC–MS

The intracellular free amino acids were obtained after the samples were freeze-dried with lyophilizer (Four-Ring Science Instrument Plant Beijing Co., Ltd., China) (Feng et al. 2012). Intracellular free amino acids were dissolved with 100 µL pyridine at 50 °C for 2 h and then derivatized with 100 µL *N*-tert-butyldimethylsilyl-*N*-methyltrifluoroacetamide (with 1% tertbutyl dimethyl chlorosilane, Sigma-Aldrich) at 60 °C for 1 h (Wittmann 2007). The separation and ^13^C-labeling pattern analysis of amino acids were analyzed by gas chromatography-mass spectrometry (Agilent 5975YC Series, Agilent Technologies, USA) (Feng et al. 2012). The split ratio was 1:10, the injection volume was 1 µL, and the carrier gas helium flow rate was 1 mL/min. The GC temperature program was as follows: hold at 100 °C for 4 min, increase at 5 °C/min to 200 °C, increase at 10 °C/min to 300 °C, and then hold for 10 min.

### Determination of the pool sizes of intracellular metabolites by LC–MS/MS

The samples used for the quantification of intracellular metabolites were obtained from the 5 L bioreactor during the exponential growth phase. The extraction process has been introduced above. To accurately determine the metabolites concentration, isotope dilution mass spectrometry (IDMS) method (Wu et al. 2005) was used. Extracted metabolites were resuspended in 1 mL deionized water, and 200 µL of ^13^C-labeled intracellular metabolites were added as internal standards. The uniformly ^13^C-labeled metabolites were extracted from the *P. pastoris* biomass cultivated in a fed-batch fermentation using uniformly ^13^C-labeled glucose as sole substrate. The obtained labeled cell extract contained the whole yeast metabolome. After internal standards added, samples were injected into a liquid chromatography (Waters Acquity T3 column (Waters Corporation, Milford, MA) with dimensions 150 mm × 2.1 mm × 1.8 mm was used for organic acids and sugar phosphates, ACQUITY UPLCR BEH Amide (Waters) 1.7 μm × 2.1 × 100 mm for nucleosides, Xselect™ HSS T3 1.8 μm × 2.1 × 100 mm Column for amino acids) coupled to a Thermo TSQ Quantum Ultra triple quadrupole instrument (Thermo Fisher Scientific) with electrospray ionization. The spray voltage, vaporizer temperature, sheath gas pressure, ion sweep gas pressure, aux gas pressure, capillary temperature used in the detection of amino acids were +3500 V, 300 °C, 15, 0, 10 psi, and 300 °C, respectively. The parameters for organic acids, nucleosides, and sugar phosphate detection were −3000 V, 200 °C, 15, 0, 10 psi, and 200 °C, respectively.

### Determination of intracellular redox cofactors

The determinations of NADH, NAD^+^, NADPH, and NADP^+^ were conducted with an Enzy Fluo™ Assay Kit (Bio Assay Systems Corporation). About 1 mL broth was obtained by the rapid-sampling setup, then quenched with 10 mL quench solution (60% methanol, −40 °C). The supernatant was discarded after centrifugation at −4 °C and 6000 rpm. The remaining processes were referred to the standard specifications. All determinations above were in triplicates.

### Isotopically instationary ^13^C metabolic flux estimation

The metabolic model used was similar to our previous study (Nie et al. 2014). However, the unidirectional reaction from α-ketoglutarate (αkG) to glutamate was changed to reversible reaction to acknowledge the uptake of glutamate. The model (Additional file 1) contains the main pathways of central carbon metabolism, including the anaplerotic pathways catalyzed by phosphoenolpyruvate carboxykinase and pyruvate carboxylase. The flux calculation was conducted by isotopomer network compartmental analysis (INCA) (Young 2014). The estimated fluxes were considered acceptable only when the obtained minimal weighted residual was below the χ^2^ at a 95% confidence level. 95% confidence intervals were computed for all the estimated fluxes by evaluating the sensitivity of the minimized sum of squared residuals (SSR) to flux variations.

## Results and discussion

### Physiology of G1HL on Glc/Glu and on Glc

**Fig. 1:**
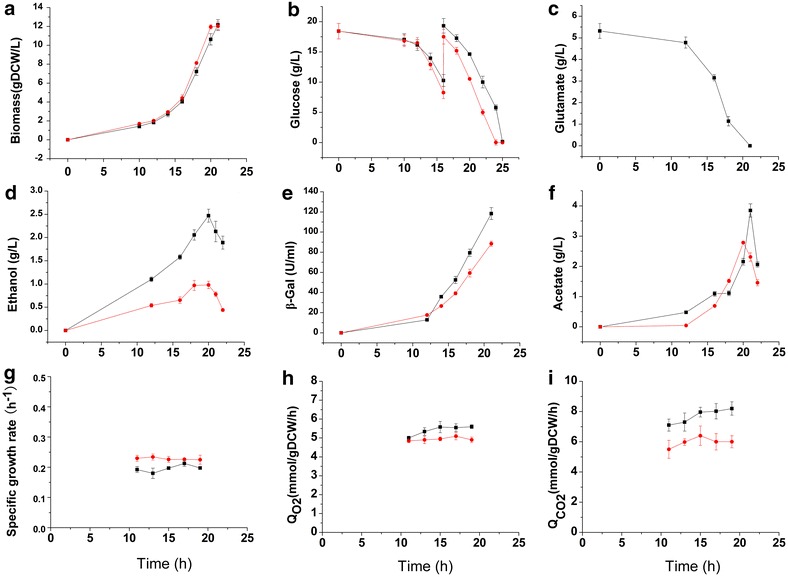
Physiological profiles of *P. pastoris* G1HL on Glc/Glu (*black line*) and on Glc (*red line*). The parameters include biomass (**a**), glucose (**b**, the *red arrow* is the time 13C glucose was added), glutamate (**c**), ethanol (**d**), β-gal (**e**), acetate (**f**), specific growth rate at the exponential phase (**g**), Q_O2_ (**h**), and Q_CO2_ (**i**). The results represent the average and the standard error of three independent samples

The addition of glutamate resulted in remarkable impacts on the physiology of the high β-galactosidase-producing strain *P. pastoris* G1HL (Fig. 1; Additional file 2). The specific rate of β-galactosidase production was 32% higher, while the specific rate of cell growth and the yield coefficient of biomass from carbon source were 6 and 15% lower on Glc/Glu than on Glc, respectively. It indicates that the improvement of β-galactosidase production was at the cost of the slightly decrease in cell growth. The reduced specific growth rate indicated the presence of metabolic burden on Glc/Glu. However, the biomass yield from glucose (*Y*_*x*/glucose_) remains almost unaltered (0.37 vs. 0.36 g DCW/g glucose on Glc and on Glc/Glu). We also found that the specific O_2_ uptake rate (Q_O2_) and the yield coefficient of β-galactosidase from oxygen was 14.5 and 14% higher on Glc/Glu than on Glc, respectively. In order to elucidate the impacts of glutamate on the central metabolism including the energy and reductive cofactor metabolism in detail, it is necessary to quantify the pool sizes of intracellular metabolites and the metabolic flux distributions on these two cultivation conditions.

### Intracellular metabolite pool

The pool sizes of amino acids, organic acids, and sugar phosphates in GHL on Glc, in G1HL on Glc, and in G1HL on Glc/Glu were quantified via IDMS method. The extracted metabolites from *P. pastoris* GHL biomass grown on uniformly ^13^C-labeled glucose as sole carbon source are used as the internal standards.

### Intracellular amino acids

Intracellular amino acids are the main building-blocks for synthesis of recombinant proteins, so the pool sizes of amino acids are undoubtedly important to learn the details of cell metabolic adaptations during recombinant protein expression. Eighteen amino acids were determined with the present method (Table 1). We observed a significant difference in the total amino acid pool size. The total amino acid pool size was 66.5% lower (102.66 vs. 306.48 µmol/gDCW) in G1HL than in GHL on Glc, but the addition of glutamate to G1HL restored the total amino acid pool size to an even higher level than that in GHL (329.16 vs. 306.48 µmol/gDCW). It indicated that the supply of amino acids was one bottleneck for high β-galactosidase production in G1HL, and the addition of glutamate released this limitation.

**Table 1:** The pool sizes of intracellular amino acids in *P. pastoris* GHL and G1HL

Metabolite	GHL (µmol/gDCW)	G1HL (µmol/gDCW)		G1HL/GHL (on Glc)	*P* value of *T* test	G1HL Glu + Glc/Glc	*P* value of *T* test
	(Glc)	(Glc)	(Glc + Glu)				
Ala	1.41 ± 0.07	0.54 ± 0.30	1.86 ± 0.01	0.38	0.03	3.44	0.02
Arg	77.32 ± 1.93	25.63 ± 3.30	56.59 ± 7.92	0.33	0.00	2.21	0.01
Asp	80.33 ± 2.75	53.57 ± 1.81	85.48 ± 3.47	0.67	0.00	1.60	0.00
Asn	2.62 ± 0.29	0.25 ± 0.03	2.09 ± 0.88	0.10	0.00	8.36	0.07
Gln	36.83 ± 1.67	5.76 ± 4.52	47.87 ± 6.46	0.16	0.00	8.31	0.00
Glu	62.57 ± 2.36	9.44 ± 7.16	91.63 ± 14.42	0.15	0.00	9.71	0.00
His	5.48 ± 0.19	0.66 ± 0.40	4.64 ± 0.42	0.12	0.00	7.03	0.00
Ile	0.97 ± 0.30	0.22 ± 0.04	1.08 ± 0.47	0.23	0.05	4.91	0.09
Orn	10.02 ± 0.28	0.95 ± 0.51	4.42 ± 1.14	0.09	0.00	4.65	0.02
Ser	7.58 ± 1.29	1.51 ± 0.95	7.26 ± 1.54	0.20	0.00	4.81	0.01
Thr	3.89 ± 0.05	0.73 ± 0.49	5.21 ± 1.09	0.19	0.01	7.14	0.01
Trp	0.75 ± 0.05	0.09 ± 0.05	1.24 ± 0.41	0.12	0.00	13.78	0.04
Val	1.91 ± 0.04	0.56 ± 0.23	2.13 ± 0.34	0.29	0.01	3.80	0.00
Leu	0.90 ± 0.27	0.21 ± 0.06	0.81 ± 0.25	0.23	0.04	3.86	0.05
Lys	3.62 ± 0.29	0.42 ± 0.17	2.51 ± 0.56	0.12	0.00	5.98	0.02
Met	0.55 ± 0.20	0.29 ± 0.03	0.65 ± 0.14	0.53	0.15	2.24	0.04
Phe	0.50 ± 0.24	0.17 ± 0.04	0.48 ± 0.20	0.34	0.14	2.82	0.11
Pro	9.24 ± 0.52	1.66 ± 1.38	13.22 ± 4.82	0.18	0.01	7.96	0.04
Total	306.48 ± 12.80	102.66 ± 21.46	329.16 ± 44.53	0.33	0.00	3.21	0.05

18 amino acids were detected. Samples were taken in the exponential growth phase, all detections were in triplicates. The significant levels of differences between GHL and G1HL grown on Glc, G1HL grown on Glc/Glu and on Glc are evaluated by two-tailed *T* test

Among the amino acids determined, glutamate is an important precursor for β-galactosidase biosynthesis (Craven et al. 1965). Nine amino acids including ASP,GLY, GLN, HIS,LYS,PRO,SER,TYR, and VAL are derived from glutamate in *P. pastoris*, thus the addition of glutamate tend to improve the recombinant protein expression. The added glutamate may be used to provide amino group for the synthesis of other amino acids by aminotransferase reaction. From flux results (Additional file 3), it can be observed that the fluxes of SER and ASP synthesis increased by 163 and 49%, respectively. The pool size of SER and ASP increased by 381% and by 60%, respectively (Table 1). The changes in amino acid pool sizes, however, varied with different degrees. Because different amino acid families are derived from different precursors, the amino acid pool size may depend on the precursor pools in some way. For example, the pool sizes of Asp and Glu was 1.6-fold (85.48 vs. 53.57 µmol/gDCW) and 9.7-fold (91.63 vs. 9.44 µmol/gDCW) higher on Glc/Glu than on Glc, respectively. The amino acid with minimum increment value should become the limitation for protein expression improvement, and therefore only about 1.5 times higher of β-galactosidase production was observed on Glc/Glu.

### Intracellular organic acids and sugar phosphates

The pool sizes of most intracellular organic acids and sugar phosphates (Table 2) were lower in G1HL than in GHL on Glc and showed different trends after the addition of glutamate.

**Table 2:** The pool size of intracellular organic acids and surge phosphates in *P. pastoris* GHL and G1HL

Metabolite	GHL (µmol/gDCW)	G1HL (µmol/gDCW)		G1HL/GHL (on Glc)	*P* value of *T* test	G1HL Glu + Glc/Glc	*P* value of *T* test
	(Glc)	(Glc)	(Glc + Glu)				
G1P	0.86 ± 0.30	0.37 ± 0.08	0.32 ± 0.07	0.43	0.10	0.86	0.46
G6P	1.84 ± 0.53	1.18 ± 0.02	1.01 ± 0.24	0.64	0.16	0.86	0.34
F6P	1.15 ± 0.87	1.56 ± 0.02	0.02 ± 0.01	1.36	0.51	0.01	0.01
FBP	0.81 ± 0.16	0.86 ± 0.01	0.22 ± 0.01	1.06	0.64	0.26	0.00
M6P	0.03 ± 0.02	0.02 ± 0.01	0.07 ± 0.01	0.67	0.50	3.50	0.00
6PGA	0.68 ± 0.33	0.26 ± 0.02	0.43 ± 0.05	0.38	0.16	1.65	0.02
PEP	0.10 ± 0.01	0.08 ± 0.01	0.11 ± 0.02	0.80	0.07	1.38	0.10
PYR	1.02 ± 0.12	0.51 ± 0.01	1.63 ± 0.68	0.50	0.02	3.20	0.10
R5P	1.81 ± 0.94	2.65 ± 1.09	0.34 ± 0.15	1.46	0.37	0.13	0.06
RL5P	9.54 ± 0.97	7.04 ± 2.51	1.47 ± 0.50	0.74	0.22	0.21	0.06
S7P	4.64 ± 2.43	2.58 ± 0.76	2.26 ± 0.53	0.56	0.28	0.88	0.59
E4P	0.20 ± 0.02	0.16 ± 0.03	0.29 ± 0.53	0.80	0.14	1.81	0.01
αKG	0.35 ± 0.03	0.02 ± 0.01	0.57 ± 0.37	0.06	0.00	28.50	0.12
Cit	1.17 ± 0.16	0.89 ± 0.02	1.23 ± 0.39	0.76	0.09	1.38	0.27
Fum	0.21 ± 0.03	0.16 ± 0.08	0.61 ± 0.17	0.76	0.40	3.81	0.03
Mal	0.56 ± 0.61	0.49 ± 0.28	3.09 ± 0.85	0.88	0.87	6.31	0.02
Suc	0.59 ± 0.07	0.25 ± 0.17	1.02 ± 0.39	0.42	0.06	4.08	0.06

All detections were in triplicates. The significant levels of differences between GHL and G1HL grown on Glc, G1HL grown on Glc/Glu, and on Glc are evaluated by two-tailed *T* test

Most glycolytic intermediates decreased in G1HL compared to GHL, which was linked to the lower glucose uptake rate and glycolytic flux (Nie et al. 2014). The addition of glutamate increased the pool sizes of PEP and PYR, while the others still remained at low levels.

The intermediate metabolites in PP pathway are the main precursors for the synthesis of DNA, RNA, and some aromatic amino acids. The pool sizes of most PPP intermediates in G1HL also decreased compared to GHL. After the addition of glutamate, the pool size of 6-phosphogluconate (6PGA) and erythrose-4-phosphate (E4P) increased. Since E4P is the main precursor of aromatic amino acids, the increase in the pool size of E4P was related to the increased need of these aromatic amino acids for high β-galactosidase production.

The pool sizes of all TCA cycle intermediates were lower in G1HL than in GHL on Glc. The pool size of αKG in G1HL had the most obvious decrease in the intermediates of TCA cycle. αKG is one of the main precursors of amino acids; the high β-galactosidase expression resulted in the decrease in amino acids pool size, which in turn declined the pool size of αKG. After the addition of glutamate, the pool sizes of TCA cycle intermediates were much higher than those on Glc. The formation of αKG from glutamate is the first step to use glutamate through TCA cycle. The αKG pool size was about 25-fold higher (0.57 vs. 0.02 μmol/gDCW) on Glc/Glu than on Glc. The increased supply of αKG pool thus improved the pool sizes of other metabolites in TCA cycle.

### Isotopically instationary ^13^C-MFA

**Fig. 2:**
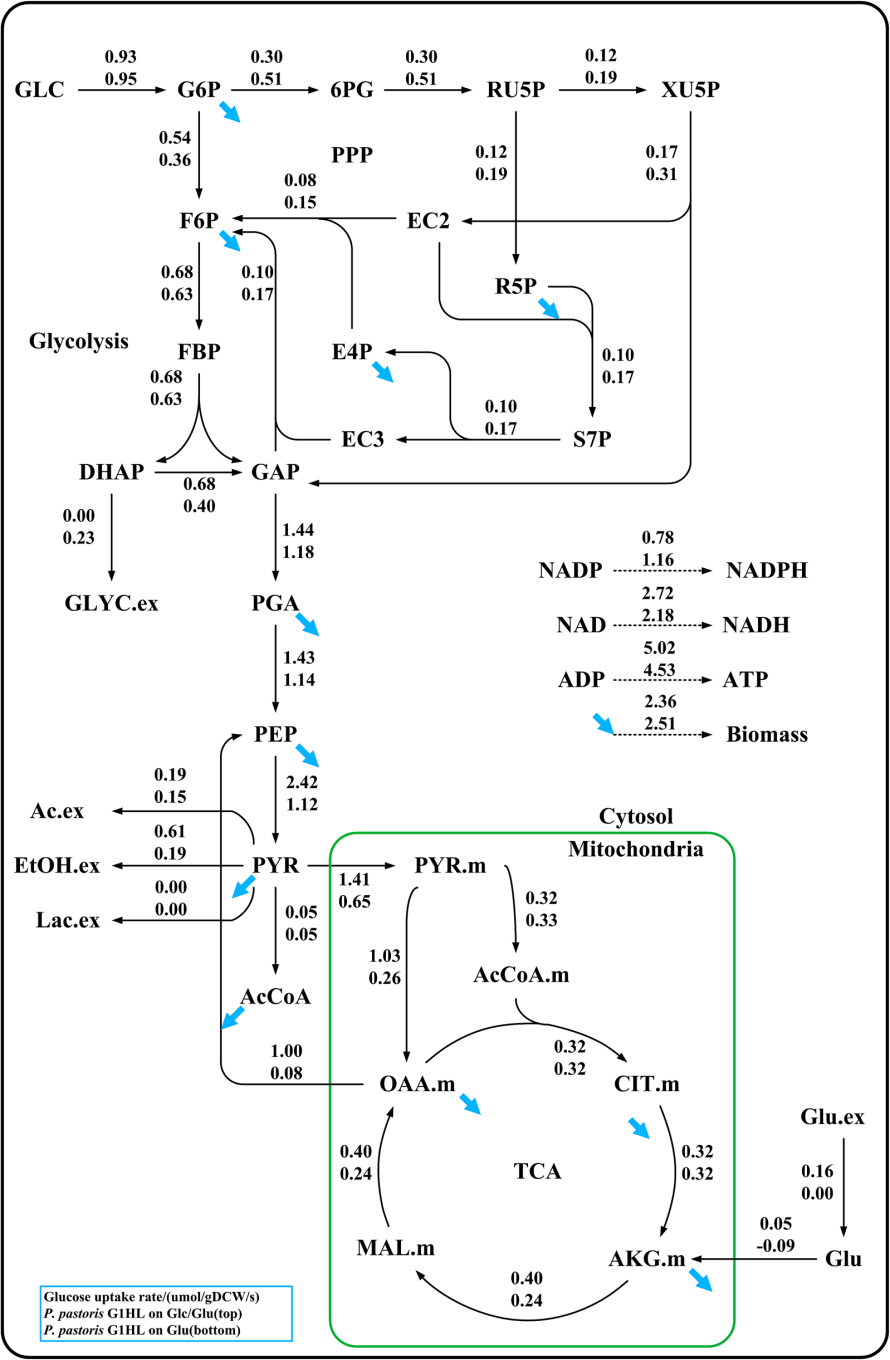
Flux distributions in *P. pastoris* G1HL during the exponential growth phase on Glc/Glu and Glc. The *blue arrows* represent the fluxes for biomass synthesis. For flux estimation, ten samples were taken within 5 min

The carbon flux distributions of G1HL on Glc/Glu and on Glc were quantified via isotopically instationary ^13^C-MFA. The rates of substrate consumption, biomass growth, β-galactosidase production, CO_2_, and by-products formation were determined and the carbon recovery were confirmed for both conditions (Table 3), indicating that the metabolic flux analysis was feasible. The metabolic fluxes were estimated based on the dynamics of the mass isotopomer distributions (MID) of intracellular free amino acids (Additional file 4) and the measured metabolite pool sizes. The estimated fluxes (Fig. 2) were statistically acceptable due to the fact that the SSR values were in the expected ranges (221.5 in [161.8, 240.0] on Glc and 212.7 in [115.5, 232.3] on Glc/Glu) (Additional file 5).

**Table 3:** The carbon recovery during isotopic labeling experiments

Substrate	Glucose (mmolC/gDCW/h)	Glutamate (mmolC/gDCW/h)	Biomass (mmolC/gDCW/h)	CO_2_ (mmolC/gDCW/h)	By-product (mmolC/gDCW/h)	Recovery (%)
Glc	20.52 ± 0.12	0	9.04 ± 0.04	5.96 ± 0.05	4.93 ± 0.34	97.19 ± 1.52
Glc/Glu	19.98 ± 0.12	2.81 ± 0.25	8.49 ± 0.12	7.96 ± 0.07	5.76 ± 0.21	97.47 ± 0.17

The flux of EMP entry reaction catalyzed by phosphoglucoiomerase (PGI) was 50% higher on Glc/Glu than on Glc (0.54 vs. 0.36 µmol/gDCW/s). This up-regulation was related to the increased ATP demand for higher β-galactosidase expression (Jan Heyland et al. 2010) which will be discussed in “Possible modulation mechanism of glutamate” section. The flux of the next reaction catalyzed by phosphofructokinase (PFK), however, was merely 7.9% higher (0.68 vs. 0.63 µmol/gDCW/s). We have found in “Intracellular organic acids and Sugar phosphates” section that the pool sizes of PYR increased on Glc/Glu, which exerted a feedback inhibition on PFK (Guminska and Wazewska-Czyzewska 1975) and therefore weakened the increase degree of PFK flux on Glc/Glu. Furthermore, the ethanol production was higher with glutamate addition as show in Fig. 1g. It seems that the higher ethanol production may be caused by the combined effect of higher pyruvate kinase flux (2.42 vs. 1.12 μmmol/gDCW/s), higher PYR pool size (1.63 vs. 0.51 μmmol/gDCW), and the unaltered upstream TCA cycle flux (0.32 vs. 0.33 μmmol/gDCW/s), which result in overflow metabolism at PYR note.

The flux of PPP oxidative branch on Glc/Glu was about 40% lower than that on Glc (0.30 vs. 0.51 µmol/gDCW/s). The oxidative branch of PPP is the main pathway for cytosolic NADPH production. The flux through this pathway is generally related to the biosynthetic demand for NADPH (Blank et al. 2005). Although higher β-galactosidase production was observed on Glc/Glu, the fraction of β-galactosidase to the total cell protein was very small (the amount of intracellular β-galactosidase was 0.128 and 0.165 mg/gDCW on Glc and on Glc/Glu, respectively). Thus the decrease in the flux in PPP oxidative branch seems to be related to the efficiency of glucose utilization and the specific growth rate, but not to the increased NADPH supply.

The fluxes in upstream TCA reactions (from OAA to αKG) remained unaltered, and the fluxes in downstream TCA reactions (from αKG to OAA) were 67% higher on Glc/Glu than on Glc. About 30% glutamate assimilated was streamed into the TCA cycle, while the rest was used as building-block precursors to the biosynthesis of amino acids and β-galactosidase. The almost unchanged fluxes in upstream TCA reactions showed the robustness of fluxes in these reactions. Sonenshein and Toya (2007); Toya et al. (2014) also observed similar phenomena in their studies of the effects of glutamate on the metabolism of *Bacillus subtilis*.

### Redox cofactors and energy metabolism

Redox cofactors (NADPH and NADH) and energy (ATP) play pivotal roles (Jordà et al. 2012; Parekh and Wittrup 1997) in recombinant protein expression. Among thousands of metabolic reactions, cofactors participate in about 300 oxidation and reduction reactions (Sauer et al. 2014). To investigate the impacts of glutamate on the redox cofactors and energy metabolism, we determined the pool sizes of intracellular coenzymes and nucleotides (Table 4). The production and consumption rates for NADPH, NADH, and ATP were calculated from the estimated metabolic fluxes.

**Table 4:** The pool sizes of coenzymes in *P. pastoris* G1HL on Glc and on Glc/Glu

Metabolite	Glc (μmol/gDCW)		Glc/Glu (μmol/gDCW)		Glc + Glu/Glc	*P* value of *T* test
	Value	SD	Value	SD		
ADP	0.19	0.09	0.61	0.25	3.12	0.09
AMP^a^	0.55	0.06	2.38	0.72	4.36	0.05
CAMP	0.31	0.27	0.35	0.27	1.10	0.89
CMP	0.26	0.02	13.61	8.12	52.97	0.10
GMP^a^	0.19	0.07	0.67	0.18	3.48	0.03
UMP	0.34	0.15	0.54	0.06	1.61	0.13
NAD^+a^	0.27	0.00	0.91	0.04	3.33	0.00
NADH^a^	0.15	0.01	0.32	0.04	2.13	0.01
NADP^+a^	0.88	0.02	1.00	0.01	1.13	0.00
NADPH^a^	0.60	0.05	0.77	0.06	1.29	0.03

^a^The pool sizes of metabolites in G1HL on Glc/Glu were significantly up-regulated or down-regulated compared to those on Glc by two-tailed *T* test with *P* = 0.05

**Fig. 3:**
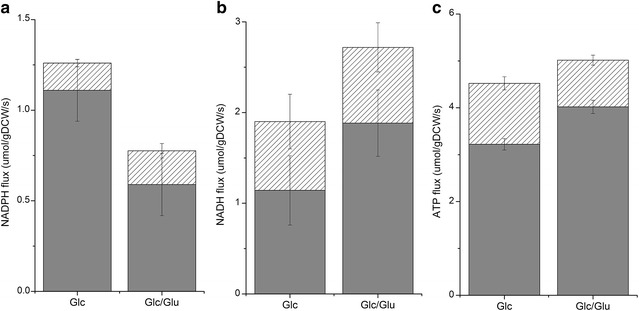
Regeneration fluxes of energy and redox cofactors on Glc/Glu and on Glc. **a** NADPH regeneration flux. *Gray bar* by oxidative PPP oxidative branches; *Hatched bar* by AADH pathway. **b** NADH regeneration flux. *Gray bar* by TCA pathway; *Hatched bar* by EMP cycle. **c** Total ATP regeneration flux. *Gray bar* by oxidative phosphorylation (the efficiency of oxidative phosphorylation is 1.48 mol ATP/mol O); *Hatched bar* by substrate level phosphorylation

In *P. pastoris*, there are two routes for NADPH generation, the oxidative branch of PPP and the acetate formation pathway catalyzed by acetaldehyde dehydrogenase (AADH) (Nocon et al. 2014), which can use NAD^+^ and/or NADP^+^ as cofactor (Blank et al. 2005). Assuming that AADHs only used NADPH as cofactor, the NADPH generation rates from oxidative PPP and AADHs were calculated (Fig. 3a). We noticed that the NADPH generation rate was negatively related to the β-galactosidase production rate. This result is contrary to what was reported previously for G1HL and for other recombinant protein-producing strains, e.g., *Schizosaccharomyces pombe* secreting maltase (Klein et al. 2014), *Aspergillus niger* secreting fructofuranosidase (Driouch et al. 2012), *Saccharomyces cerevisiae* producing superoxide dismutase (Gonzalez et al. 2003), and *P. pastoris* producing human superoxide dismutase (Nocon et al. 2014). However, in the work of Driouch, the authors speculated that the activation of PPP and malic enzyme even generated an apparent excess for NADPH, and it could be due to the use of extra NADPH for protein folding and possible ER stress in connection with secreting the recombination protein. In the present study, the decreased NADPH generation still met the requirement for higher β-galactosidase expression without the secreting process, which indicated that the high β-galactosidase production in G1HL was not NADPH limited. This phenomenon showed that the effect of higher recombinant protein expression on PP pathway depends not only on the host species, but also on the produced protein and even on the extracellular environments.

NADH, as an important redox cofactor for cell growth and recombinant protein expression, plays a key role in the maintenance of redox balance and energy generation (Hou et al. 2009). We found that almost two-thirds of NADH was derived from TCA cycle on both conditions, indicating that the TCA cycle was the main pathway for NADH generation (Fig. 3b). The net generation rate of NADH was about 25% higher on Glc/Glu than on Glc (2.72 vs. 2.18 μmol/gDCW/s) correlating with the higher fluxes in downstream TCA cycle. The pool sizes of NADH and NAD^+^ were measured with Enzy Fluo™ Assay Kit (Table 4), and the NADH/NAD^+^ ratio on Glc/Glu was lower than that on Glc (0.35 vs. 0.55), indicating that the cells on Glc/Glu were in a less reducing condition.

ATP is the energy currency in cells and mainly generated by two ways, the oxidative phosphorylation and the substrate-level phosphorylation. The ATP generation rate through oxidative phosphorylation was calculated assuming that all the NADH was recycled through the respiratory chain and the efficiency of oxidative phosphorylation (P/O ratio) was 1.48 mol ATP/molar O (Verduyn 1991). We found that the oxidative phosphorylation was the main route for ATP generation on both conditions (Fig. 3c), and the percentage of ATP from oxidative phosphorylation was 12% higher on Glc/Glu than on Glc (80 vs. 71%). The reactions in the downstream of TCA cycle (from αKG to OAA) can derive 3 mol NADH and 1 mol ATP per molar αKG consumed and the fluxes in this part were 67% higher on Glc/Glu, indicating that the increased fluxes in downstream TCA cycle were crucial for higher energy generation and consequently higher β-galactosidase production.

ATP is consumed for cellular maintenance, biomass synthesis, and production (Chung et al. 2010). Protein biosynthesis is an energy-intensive process, in which 4 mol ATP is consumed per molar bond formed (Klein et al. 2014). The extra ATP demand caused by heterologous protein expression is known as metabolic burden, which can be quantified by the yield of biomass from ATP (Heyland et al. 2011). We found that the yield coefficient of biomass from ATP was 15% lower on Glc/Glu than on Glc (11.84 vs. 13.93 g DCW/mol ATP), indicating a higher metabolic burden on Glc/Glu. It has been reported that the metabolic burden in yeasts is caused by the posttranslational processes (folding and secretion) and/or the degradation of intact or unfolded proteins in the endoplasmic reticulum (Mattanovich et al. 2004). To overcome this metabolic burden, the *P. pastoris* cell adjusts the flux distribution, which consequently enables a high yield of ATP from glucose (Heyland et al. 2011). The ATP yield from glucose was indeed 13% higher on Glc/Glu than on Glc (5.39 vs. 4.76 mol ATP/mol glucose). Jordà and co-workers (Jordà et al. 2014) have studied the impact of metabolic burden on trehalose cycle in Rol-producing *P. pastoris* by metabolomics and ^13^C-MFA and found that the stress-related trehalose pool size and trehalose recycling flux all increase in the Rol-producing strain compared to control strain. The trehalose recycling is a futile cycle, which could contribute to the increased energy demand and lower biomass yield.

The expression of recombinant protein may exert an impact on energy charge. Although the pool size of ATP was hard to be quantified precisely due to its rapid turnover, we can assess the energy status on two conditions. It has been reported that the adenine nucleotide pools are equilibrated by the myokinase reaction during oscillatory glycolysis (Das and Busse 1992). Based on this hypothesis and the fact that the much higher pool size of ADP and AMP on Glc/Glu, we inferred that the the ratio of ATP/AMP was lower on Glc/Glu than on Glc.

### Possible modulation mechanism of glutamate

**Fig. 4:**
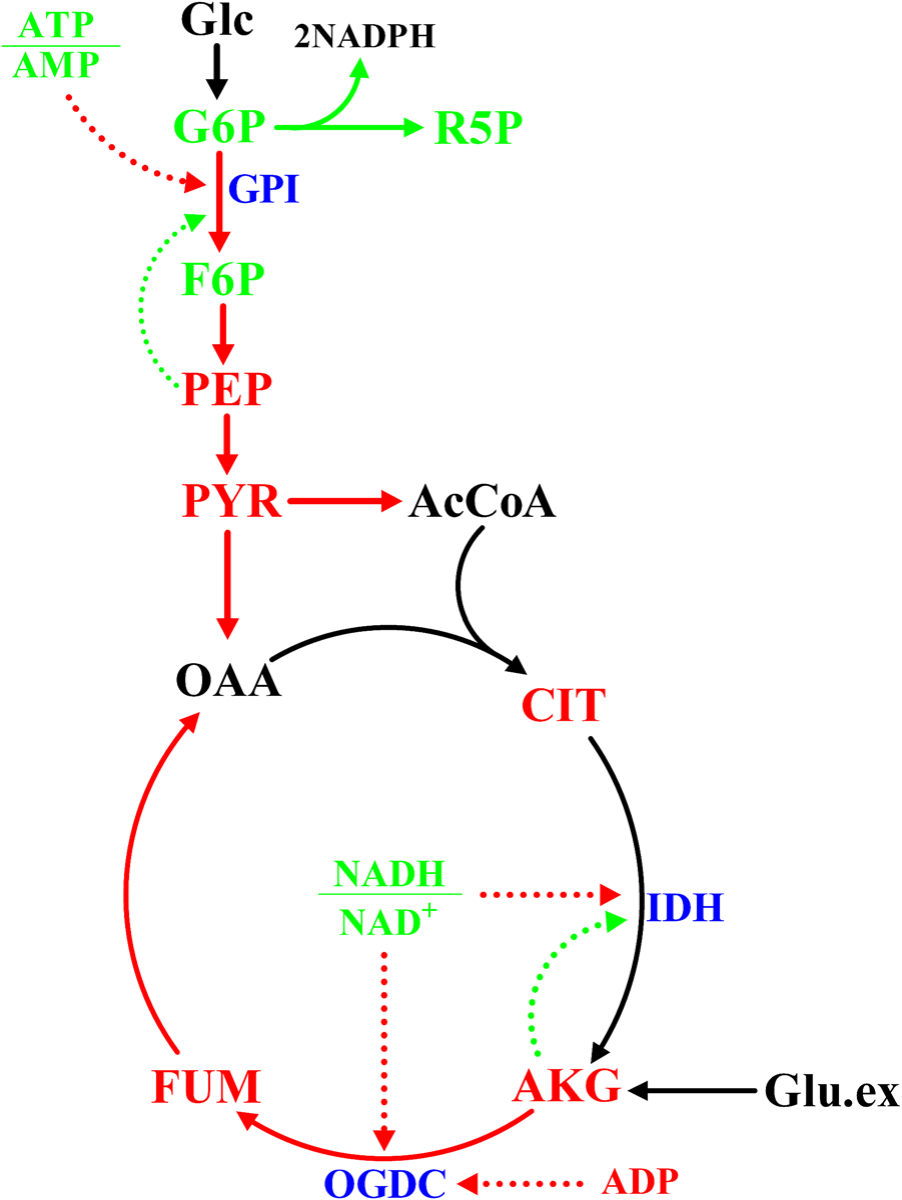
Possible modulation mechanism of glutamate on the central metabolism of *P. pastoris* G1HL. *Red solid lines* up-regulated fluxes, *green solid lines* down-regulated fluxes. *Red metabolite names* metabolites with increased pool sizes, *green metabolite names* metabolites with decreased pool sizes. *Red dotted lines* positive regulations, *green dotted lines* negative regulations. (*GPI* glucose-6-phosphate isomerase; *IDH* isocitrate dehydrogenase; *OGDC* α-ketoglutaric dehydrogenase)

We inferred that there exists an intricate modulation mechanism in *P. pastoris* G1HL to adapt to the addition of glutamate (Fig. 4). The glutamate addition increased the pool sizes of intracellular organic acids and amino acids, thus releasing the limitation of amino acids as building-blocks for high β-galactosidase production. The increased β-galactosidase production required more redox cofactors and energy. To meet these demands, the fluxes in central metabolism were redistributed through the regulations of the metabolites in key nodes together with the energy and redox status.

In EMP pathway, the glucose-6-phosphate isomerase (GPI) is activated by the lower ratio of ATP/AMP (Takama and Nosoh 1982) and allosterically inhibited by the accumulated PEP (Ogawa et al. 2007). The effects of the activation overwhelmed that of the inhibition, thus the flux of GPI strikingly increased 50% on Glc/Glu. For the isocitrate dehydrogenase (IDH), there is the feedback inhibition of high αKG pool size (Toya et al. 2014) and the activation of low ratio value of NADH to NAD^+^. Furthermore, RTG genes which control the pathway of αKG synthesis can be repressed by glutamate (Liu and Butow 1999). Thus the interaction of these factors kept the flux in upstream TCA cycle unaltered on Glc/Glu. For the α**-**ketoglutaric dehydrogenase (OGDC), the increased ADP pool size and the lower NADH/NAD^+^ ratio can enhance the enzyme activity, so the OGDC flux increased 67%. Procopio has studied the effect of amino acid supplementation on the transcription of *Saccharomyces pastorianus* and found the up-regulation of the pyruvate decarboxylases (Procopio et al. 2015), which is in accordance with the increased ethanol generation flux on Glc/Glu in present work. Thus the intracellular metabolic flux status can also reflect the modulation mechanism in the transcription level. More evidences from multi-omics studies, however, are required to clarify the question whether the key enzymes for the EMP and TCA pathways are transcriptionally regulated or not.

## Conclusion

The study aimed at revealing the impacts of glutamate on the central metabolism of the high β-galactosidase-producing strain *P. pastoris* G1HL. We found that the addition of glutamate improved β-galactosidase production and reduced cell growth and carbon source utilization efficiency. ^13^C-assisted metabolomics analysis showed that the pool sizes of intracellular amino acids were obviously higher on Glc/Glu than on Glc. The isotopically instationary ^13^C-metabolic flux analysis illustrated that the central carbon fluxes were redistributed in response to the addition of glutamate. The fluxes in EMP and in downstream TCA cycle were 50 and 67% higher on Glc/Glu than on Glc, respectively. While the fluxes in upstream TCA cycle kept almost unaltered, the fluxes in PPP oxidative branch decreased. The impacts of glutamate on the central metabolism of *P. pastoris* G1HL was inferred to be modulated through the increase and/or decrease of the metabolite concentrations in key nodes together with the energy and redox status in order to meet the increased demands of redox cofactors and energy for higher β-galactosidase production on Glc/Glu. These results enrich the understanding of the impacts of glutamate addition on central metabolism of *P. pastoris* G1HL.

## Additional files

**Additional file 1.** Stoichiometric and carbon atom transition model for *P. pastoris.*

**Additional file 2.** The physiology parameters of cultures for *P. pastoris* G1HL on Glc and on Glc/Glu.

**Additional file 3.** Estimated flux values with 95% confidence intervals in *P. pastoris* G1HL grown on Glc and Glc/Glu.

**Additional file 4.** Trends of the MID of intracellular free amino acids during ^13^C labeling experiments on Glc and on Glc/Glu.

**Additional file 5.** Measured and estimated mass isotopomer distributions of the intracellular free amino acids and the metabolite pool sizes in G1HL on Glc and Glc/Glu.

## Abbreviations

Glu: glutamate
Glc: glucose
MFA: metabolic flux analysis
IDMS: isotope dilution mass spectrometry
SSR: sum of squared residuals
β-Gal: β-Galactosidase
G1P: glucose-6-phosphate
G6P: glucose-6-phosphate
FBP: fructose-1,6-biphosphate
F6P: fructose-6-phosphate
M6P: manose-6-phosphate
6PGA: 6-phosphogluconate
PEP: phosphoenolpyruvate
PYR: pyruvate
R5P: ribose-5-phosphate
RL5P: ribulose-5-phosphate
S7P: sedoheptulose-7-phosphate
E4P: eritrose-4-phosphate
αKG: α-Ketoglutarate
Cit: citric acid
Fum: fumarate
Mal: malate
Suc: succinic acid

## References

[CR1] Blank LM, Lehmbeck F, Sauer U (2005). Metabolic-flux and network analysis in fourteen hemiascomycetous yeasts. FEMS Yeast Res.

[CR2] Canelas AB, ten Pierick A, Ras C, Seifar RM, van Dam JC, van Gulik WM, Heijnen JJ (2009). Quantitative evaluation of intracellular metabolite extraction techniques for yeast metabolomics. Anal Chem.

[CR3] Carnicer M, Canelas AB, Ten Pierick A, Zeng Z, van Dam J, Albiol J, Ferrer P, Heijnen JJ, van Gulik W (2012). Development of quantitative metabolomics for *Pichia pastoris*. Metabolomics.

[CR4] Cereghino GPL, Cereghino JL, Ilgen C, Cregg JM (2002). Production of recombinant proteins in fermenter cultures of the yeast *Pichia pastoris*. Curr Opin Biotechnol.

[CR5] Chen X, Alonso AP, Allen DK, Reed JL, Shachar-Hill Y (2011). Synergy between 13C-metabolic flux analysis and flux balance analysis for understanding metabolic adaptation to anaerobiosis in *E. coli*. Metab Eng.

[CR6] Chung B, Selvarasu S, Camattari A, Ryu J, Lee H, Ahn J, Lee D, Lee D-Y (2010). Research Genome-scale metabolic reconstruction and in silico analysis of methylotrophic yeast *Pichia pastoris* for strain improvement. Microb Cell Fact.

[CR7] Cos O, Serrano A, Montesinos JL, Ferrer P, Cregg JM, Valero F (2005). Combined effect of the methanol utilization (Mut) phenotype and gene dosage on recombinant protein production in *Pichia pastoris* fed-batch cultures. J Biotechnol.

[CR8] Craven GR, Steers E, Anfinsen CB (1965). Purification, composition, and molecular weight of the β-galactosidase of *Escherichia coli* K12. J Biol Chem.

[CR9] Cregg JM, Vedvick TS, Raschke WC (1993). Recent advances in the expression of foreign genes in *Pichia pastoris*. Nat Biotech.

[CR10] Das J, Busse H-G, Mosekilde E, Mosekilde L (1992). Analysis of the adenine nucleotide pool in an oscillating extract of yeast *Saccharomyces Uvarum*. Complexity, chaos, and biological evolution.

[CR11] De Schutter K, Lin YC, Tiels P, Van Hecke A, Glinka S, Weber-Lehmann J, Rouze P, Van de Peer Y, Callewaert N (2009). Genome sequence of the recombinant protein production host *Pichia pastoris*. Nat Biotechnol.

[CR12] Driouch H, Melzer G, Wittmann C (2012). Integration of in vivo and in silico metabolic fluxes for improvement of recombinant protein production. Metab Eng.

[CR13] Feng X, Zhuang W, Colletti P, Tang Y (2012). Metabolic pathway determination and flux analysis in nonmodel microorganisms through 13C-isotope labeling. Microb Syst Biol.

[CR14] Glick BR (1995). Metabolic load and heterologous gene expression. Biotechnol Adv.

[CR15] Gonzalez R, Andrews BA, Molitor J, Asenjo JA (2003). Metabolic analysis of the synthesis of high levels of intracellular human SOD in *Saccharomyces cerevisiae* rhSOD 2060 411 SGA122. Biotechnol Bioeng.

[CR16] Görgens JF, Passoth V, van Zyl WH, Knoetze JH, Hahn-Högerdal B (2005). Amino acid supplementation, controlled oxygen limitation and sequential double induction improves heterologous xylanase production by *Pichia stipitis*. FEMS Yeast Res.

[CR17] Görgens JF, van Zyl WH, Knoetze JH, Hahn-Högerdal B (2005). Amino acid supplementation improves heterologous protein production by *Saccharomyces cerevisiae* in defined medium. Appl Microb Biotechnol.

[CR18] Gumińska M, Ważewska-Czyżewska M (1975). Enzymatic pattern of glucose metabolic pathways in pyruvate kinase-deficient erythrocytes. Clin Chim Acta.

[CR19] Hamilton SR, Davidson RC, Sethuraman N, Nett JH, Jiang Y, Rios S, Bobrowicz P, Stadheim TA, Li H, Choi B-K (2006). Humanization of yeast to produce complex terminally sialylated glycoproteins. Science.

[CR20] Heyland J, Fu J, Blank LM, Schmid A (2011). Carbon metabolism limits recombinant protein production in *Pichia pastoris*. Biotechnol Bioeng.

[CR21] Hou J, Lages NF, Oldiges M, Vemuri GN (2009). Metabolic impact of redox cofactor perturbations in *Saccharomyces cerevisiae*. Metab Eng.

[CR22] Jan Heyland JF, Blank Lars M, Schmid A (2010). Quantitative physiology of *Pichia pastoris* during glucose-limited high-cell density fed-batch cultivation for recombinant protein production. Biotechnol Bioeng.

[CR23] Jordà J, Jouhten P, Cámara E, Maaheimo H, Albiol J, Ferrer P (2012). Metabolic flux profiling of recombinant protein secreting *Pichia pastoris* growing on glucose: methanol mixtures. Microb Cell Fact.

[CR24] Jordà J, Rojas HC, Carnicer M, Wahl A, Ferrer P, Albiol J (2014). Quantitative metabolomics and instationary 13C-metabolic flux analysis reveals impact of recombinant protein production on trehalose and energy metabolism in *Pichia pastoris*. Metabolites.

[CR25] Klein T, Lange S, Wilhelm N, Bureik M, Yang TH, Heinzle E, Schneider K (2014). Overcoming the metabolic burden of protein secretion in *Schizosaccharomyces pombe*-a quantitative approach using 13C-based metabolic flux analysis. Metab Eng.

[CR26] Liu Z, Butow RA (1999). A transcriptional switch in the expression of yeast tricarboxylic acid cycle genes in response to a reduction or loss of respiratory function. Mol Cell Biol.

[CR27] Macauley-Patrick S, Fazenda ML, McNeil B, Harvey LM (2005). Heterologous protein production using the *Pichia pastoris* expression system. Yeast.

[CR28] Mattanovich D, Gasser B, Hohenblum H, Sauer M (2004). Stress in recombinant protein producing yeasts. J Biotechnol.

[CR29] Nie Y, Huang M, Lu J, Qian J, Lin W, Chu J, Zhuang Y, Zhang S (2014). Impacts of high β-galactosidase expression on central metabolism of recombinant *Pichia pastoris* GS115 using glucose as sole carbon source via 13C metabolic flux analysis. J Biotechnol.

[CR30] Niu H, Jost L, Pirlot N, Sassi H, Daukandt M, Rodriguez C, Fickers P (2013). A quantitative study of methanol/sorbitol co-feeding process of a *Pichia pastoris* Mut+/pAOX1-lacZ strain. Microb Cell Fact.

[CR31] Nocon J, Steiger MG, Pfeffer M, Sohn SB, Kim TY, Maurer M, Russmayer H, Pflugl S, Ask M, Haberhauer-Troyer C, Ortmayr K, Hann S, Koellensperger G, Gasser B, Lee SY, Mattanovich D (2014). Model based engineering of *Pichia pastoris* central metabolism enhances recombinant protein production. Metab Eng.

[CR32] Ogawa T, Mori H, Tomita M, Yoshino M (2007). Inhibitory effect of phosphoenolpyruvate on glycolytic enzymes in *Escherichia coli*. Res Microbiol.

[CR33] Parekh RN, Wittrup KD (1997). Expression level tuning for optimal heterologous protein secretion in *Saccharomyces cerevisiae*. Biotechnol Prog.

[CR34] Procopio S, Sprung P, Becker T (2015). Effect of amino acid supply on the transcription of flavour-related genes and aroma compound production during lager yeast fermentation. LWT Food Sci Technol.

[CR35] Qin X, Qian J, Yao G, Zhuang Y, Zhang S, Chu J (2011). GAP promoter library for fine-tuning of gene expression in *Pichia pastoris*. Appl Environ Microbiol.

[CR36] Sauer M, Branduardi P, Rußmayer H, Marx H, Porro D, Mattanovich D, Piškur J, Compagno C (2014). Production of metabolites and heterologous proteins. Molecular mechanisms in yeast carbon metabolism.

[CR37] Sonenshein AL (2007). Control of key metabolic intersections in *Bacillus subtilis*. Nat Rev Microbiol.

[CR38] Takama M, Nosoh Y (1982). Effect of ATP on glucose-6-phosphate isomerase from *Bacillus caldotenax*. Biochim Biophys Acta.

[CR39] Toya Y, Hirasawa T, Morimoto T, Masuda K, Kageyama Y, Ozaki K, Ogasawara N, Shimizu H (2014). 13C-metabolic flux analysis in heterologous cellulase production by *Bacillus subtilis* genome-reduced strain. J Biotechnol.

[CR40] Verduyn C (1991). Physiology of yeasts in relation to biomass yields. Antonie Van Leeuwenhoek.

[CR41] Wasylenko TM, Stephanopoulos G (2015). Metabolomic and 13C-metabolic flux analysis of a xylose-consuming *Saccharomyces cerevisiae* strain expressing xylose isomerase. Biotechnol Bioeng.

[CR42] Wittmann C (2007). Fluxome analysis using GC-MS. Microb Cell Fact.

[CR43] Wu L, Mashego MR, van Dam JC, Proell AM, Vinke JL, Ras C, van Winden WA, van Gulik WM, Heijnen JJ (2005). Quantitative analysis of the microbial metabolome by isotope dilution mass spectrometry using uniformly 13C-labeled cell extracts as internal standards. Anal Biochem.

[CR44] Young JD (2014). INCA: a computational platform for isotopically non-stationary metabolic flux analysis. Bioinformatics.

[CR45] Zamboni N, Fendt S-M, Ruhl M, Sauer U (2009). 13C-based metabolic flux analysis. Nat Protoc.

