# Supplementary material for: Combined 13C-assisted metabolomics and metabolic flux analysis reveals the impacts of glutamate on the central metabolism of high β-galactosidase-producing Pichia pastoris
Source: Bioresour Bioprocess. 2016 Nov 2;3(1):47. doi: 10.1186/s40643-016-0124-6 (PMC5093185; doi:10.1186/s40643-016-0124-6)
Supplement: Supplementary file 1 — Additional file 1. Stoichiometric and carbon atom transition model for P. pastoris. [file 40643_2016_124_MOESM1_ESM.doc]

1. **Stoichiometric and carbon atom transition model for *P. pastoris***

| Name | Equation |
| --- | --- |
| upt_glucose | GLC.ex (abcdef) → GLC (abcdef) |
| emp1 | GLC (abcdef ) + ATP → G6P (abcdef) + ADP |
| emp2 | G6P (abcdef) ↔ F6P (abcdef) |
| emp3 | F6P (abcdef)+ ATP ↔ FBP (abcdef) |
| emp4a | FBP (abcdef) → DHAP (cba) + GAP (def) |
| emp4b | DHAP (abc) + NADH → GLYC (abc) + NAD |
| emp5 | DHAP (abc) ↔ GAP (abc) |
| emp6 | GAP (abc) + NAD + ADP ↔ PGA (abc) + NADH + ATP |
| emp7 | PGA (abc) ↔ PEP (abc) |
| emp8 | PEP (abc) + ADP → PYR (abc) + ATP |
| emp9a | PYR (abc) → AcEt (bc) + CO2 (a) |
| emp9b | AcEt (ab) + NADP → Ac (ab) + NADPH |
| emp9c | AcEt (ab) + NADH→ EtOH (ab) + NAD |
| ppp1 | G6P (abcdef) + 2NADP → RU5P (bcdef) + CO2 (a) + 2NADPH |
| ppp2 | RU5P (abcde) ↔ XU5P (abcde) |
| ppp3 | RU5P (abcde) ↔ R5P (abcde) |
| ta1 | XU5P (abcde) ↔ GAP (cde) + EC2 (ab) |
| ta2 | F6P (abcdef) ↔ E4P (cdef) + EC2 (ab) |
| ta3 | S7P (abcdefg) ↔ R5P (cdefg) + EC2 (ab) |
| tk1 | F6P (abcdef) ↔ GAP (def) + EC3 (abc) |
| tk2 | S7P (abcdefg) ↔ E4P (defg) + EC3 (abc) |
| tca1a | PYR (abc) + NAD → AcCoA (bc) + CO2 (a) + NADH |
| tca1b | PYR.m (abc) + NAD → AcCoA.m (bc) + CO2 (a) + NADH |
| tca2 | AcCoA.m (ab) + OAA.m (cdef) → CIT.m (fedbac) |
| tca3 | CIT.m (abcdef) → ICIT.m (abcdef) |
| tca4 | ICIT.m (abcdef) + NAD → AKG.m (abcde) + CO2 (f) + NADH |
| tca5 | AKG.m (abcde) + NAD → SUCCoA.m (bcde) + CO2 (a) + NADH |
| tca6 | SUCCoA.m (abcd) + ADP → SUC.m (1/2 abcd + 1/2 dcba) + ATP |
| tca7 | SUC.m (1/2 abcd + 1/2 dcba) + NAD ↔ FUM.m (1/2 abcd + 1/2 dcba) + NADH |
| tca8 | FUM.m (1/2 abcd + 1/2 dcba) ↔ MAL.m (abcd) |
| tca9 | MAL.m (abcd) + NAD ↔ OAA.m (abcd) + NADH |
| ana1 | OAA.m (abcd) + ATP → PEP (abc) + CO2 (d) + ADP |
| ana2 | PYR.m (abc) + CO2 (d) + ATP → OAA.m (abcd) + ADP |
| mc_pyr | PYR (abc) ↔ PYR.m (abc) |
| aa_ala | PYR.m (abc) → ALA (abc) |
| aa_asp | OAA.m (abcd) + GLU (efghi) ↔ ASP (abcd) + AKG.m (efghi) |
| aa_glu | AKG.m (abcde) ↔ GLU (abcde) |
| aa_his | R5P (abcde) + FTHF (f) + GLU (ghijk) → HIS (edcbaf) + AKG.m (ghijk) |
| aa_gly1 | SER (abc) → GLY (ab) + MEETHF (c) |
| aa_gly2 | PGA (abc) → GLY (ab) + MEETHF (c) |
| aa_ser1 | PGA (abc) + GLU (defgh) → SER (abc) + AKG.m (defgh) |
| aa_ser2 | ASP (abcd) → SER (abc) + CO2 (d) |
| aa_tyr | E4P (abcd) + PEP (efg) + PEP (hij) + GLU (klmno) → TYR (efgijabcd) + CO2 (h) + AKG.m (klmno) |
| aa_val | PYR.m (abc) + PYR.m (def) + GLU (ghijk) → VAL (abefc) + CO2 (d) + AKG.m (ghijk) |
| meethf | GLY (ab) → MEETHF (b) + CO2 (a) |
| fthf | MEETHF (a) → FTHF (a) |
| out_glyc | GLYC (abc) → GLYC.ex (abc) |
| out_ac | Ac (ab) → Ac.ex (ab) |
| out_etoh | EtOH (ab) → EtOH.ex (ab) |
| out_pyr | PYR (abc) → PYR.ex (abc) |
| out_CO2 | CO2 (abc) → CO2.ex (abc) |
| exch_CO2 | CO2 (a) ↔ CO2.env + CO2.dum (a) |
| biomass | 1.387*G6P + 0.6495*F6P + 0.2793*R5P + 0.2448*E4P + 2.689*PYR + 0.9182*OAA.m + 1.009*AKG.m + 0.0462*AcCoA.m + 0.8605*AcCoA + 0.391*MEETHF + 0.5106*ALA + 0.2397*ASP + 0.3181*GLU + 0.391*GLY + 0.1059*HIS + 0.1097*TYR + 0.3729*SER + 0.2539*VAL → 39.53*Biomass |

For condition with glutamate the reaction Glu.ex→Glu was added.

**2. Unbalanced metabolites**

Ac.ex, ADP, ATP, Biomass, CO2.env, CO2.ex, EtOH.ex, GLC.ex, GLYC.ex, NAD(P), NAD(P)H, PYR.ex, Glu.ex.
