# Supplementary material for: Combined 13C-assisted metabolomics and metabolic flux analysis reveals the impacts of glutamate on the central metabolism of high β-galactosidase-producing Pichia pastoris
Source: Bioresour Bioprocess. 2016 Nov 2;3(1):47. doi: 10.1186/s40643-016-0124-6 (PMC5093185; doi:10.1186/s40643-016-0124-6)
Supplement: Supplementary file 4 — Additional file 4. Trends of the MID of intracellular free amino acids during 13C labeling experiments on Glc and on Glc/Glu. [file 40643_2016_124_MOESM4_ESM.doc]

1. Trends of the MID of intracellular free amion acids during ^13^C labeling experiment on Glc

2. Trends of the MID of intracellular free amion acids during ^13^C labeling experiment on Glc/Glu
